# 1-[3-(2-Naphth­yl)-5-(3,4,5-trimethoxy­phen­yl)-4,5-dihydro-1*H*-pyrazol-1-yl]ethanone

**DOI:** 10.1107/S160053680801979X

**Published:** 2008-07-31

**Authors:** Zhi-Ke Lu, Hai-Lin Diao, Shen Li, Bin He

**Affiliations:** a Forestry College, GuangXi University, Nanning 530005, People’s Republic of China

## Abstract

In the title compound, C_24_H_24_N_2_O_4_, the pendant benzene and naphthalene ring systems make dihedral angles of 87.9 (3) and 19.2 (3)°, respectively, with the central pyrazoline ring. In the crystal structure, weak C—H⋯O inter­actions help to establish the packing.

## Related literature

For a related structure, see: Lu *et al.* (2006 ▶).
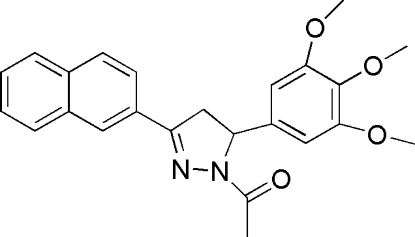


## Experimental

### 

#### Crystal data

C_24_H_24_N_2_O_4_
*M* *_r_* = 404.45
Monoclinic, 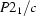

*a* = 12.611 (3) Å
*b* = 15.177 (3) Å
*c* = 10.580 (2) Å
β = 92.03 (3)°
*V* = 2023.6 (7) Å^3^
*Z* = 4
Mo *K*α radiation
μ = 0.09 mm^−1^
*T* = 113 (2) K
0.14 × 0.12 × 0.10 mm

#### Data collection

Rigaku Saturn diffractometer
Absorption correction: multi-scan (*CrystalClear*; Rigaku, 2008 ▶) *T*_min_ = 0.987, *T*_max_ = 0.991
24346 measured reflections
4655 independent reflections
3976 reflections with *I* > 2σ(*I*)
*R*_int_ = 0.040

#### Refinement

*R*[*F* ^2^ > 2σ(*F* ^2^)] = 0.048
*wR*(*F* ^2^) = 0.129
*S* = 1.07
4655 reflections
275 parameters
H-atom parameters constrained
Δρ_max_ = 0.22 e Å^−3^
Δρ_min_ = −0.28 e Å^−3^

### 

Data collection: *CrystalClear* (Rigaku, 2008 ▶); cell refinement: *CrystalClear*; data reduction: *CrystalClear*; program(s) used to solve structure: *SHELXS97* (Sheldrick, 2008 ▶); program(s) used to refine structure: *SHELXL97* (Sheldrick, 2008 ▶); molecular graphics: *SHELXTL* (Sheldrick, 2008 ▶); software used to prepare material for publication: *SHELXTL*.

## Supplementary Material

Crystal structure: contains datablocks global, I. DOI: 10.1107/S160053680801979X/hb2755sup1.cif

Structure factors: contains datablocks I. DOI: 10.1107/S160053680801979X/hb2755Isup2.hkl

Additional supplementary materials:  crystallographic information; 3D view; checkCIF report

## Figures and Tables

**Table 1:** Hydrogen-bond geometry (Å, °)

*D*—H⋯*A*	*D*—H	H⋯*A*	*D*⋯*A*	*D*—H⋯*A*
C5—H5⋯O4^i^	0.93	2.48	3.3327 (19)	152
C8—H8⋯O3^ii^	0.93	2.57	3.4320 (19)	155
C12—H12*A*⋯O1^iii^	0.97	2.55	3.3359 (18)	138
C19—H19⋯O1^iii^	0.93	2.53	3.3073 (18)	141

Symmetry codes: (i) 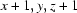; (ii) 
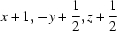
; (iii) 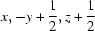.

**Fig. 1.**
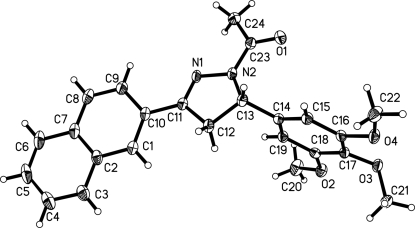
The molecular structure of (I), shown with 30% probability displacement ellipsoids (arbitrary spheres for H atoms).

## supplementary crystallographic information

### Comment

The title compound, (I), (Fig. 1) was prepared and structurally characterized as part of our ongoing studies (Lu *et al.*, 2006) of pyrazoline derivatives.

The pendant C14–C19 benzene ring and C1–C10 naphthalene ring make dihedral angles of 87.93 (6) and 19.56 (6)°, respectively, with the N1/N2/C11/C12/C13 pyrazoline ring. The dihedral angle between the benzene ring and naphthalene ring is 77.72 (6)°. Among the three methoxy groups, two are co-planar with the benzene ring, but the O3—C21 bond makes an angle of 31.3 (13)° with the ring, to minimize steric repulson between methoxy groups. The molecule of (I) is chiral: in the arbitrarily chosen asymmetric unit, C13 has *S* configuration, but crystal symmetry generates a racemic mixture.

In the crystal of (I), the molecules are linked by weak C—H···O interactions (Table 1).

### Experimental

A mixture of 1-(naphthalen-2-yl)-3-(3,4,5-trimethoxyphenyl)prop-2-en-1-one (5.0 mmol), hydrazine hydrate (25.0 mmol) and acetic acid (30 ml) was heated at reflux for 5 h, then poured onto crushed ice. The precipitate was separated by filtration, washed with water, and crystallized from trichloromethane–methanol to obtain the title compound.

The title compound (40 mg) was dissolved in mixture of acetone (10 ml) and water (10 ml) and the solution was kept at room temperature for 10 d. Natural evaporation of the solution gave colourless blocks of (I): Mp. 415–416 K.

### Refinement

All H atoms were placed geometrically (C—H = 0.93–0.98 Å), and refined as riding with *U*_iso_(H) = 1.2*U*_eq_(C) or 1.5*U*_eq_(methyl C).

### Crystal data

**Table:**

C_24_H_24_N_2_O_4_	*F*_000_ = 856
*M**_r_* = 404.45	*D*_x_ = 1.328 Mg m^−^^3^
Monoclinic, *P*2_1_/*c*	Melting point = 415–416 K
Hall symbol: -P 2ybc	Mo *K*α radiation λ = 0.71073 Å
*a* = 12.611 (3) Å	Cell parameters from 5898 reflections
*b* = 15.177 (3) Å	θ = 1.9–27.5º
*c* = 10.580 (2) Å	µ = 0.09 mm^−^^1^
β = 92.03 (3)º	*T* = 113 (2) K
*V* = 2023.6 (7) Å^3^	Block, colourless
*Z* = 4	0.14 × 0.12 × 0.10 mm

### Data collection

**Table:**

Rigaku Saturn diffractometer	4655 independent reflections
Radiation source: rotating anode	3976 reflections with *I* > 2σ(*I*)
Monochromator: confocal	*R*_int_ = 0.040
*T* = 113(2) K	θ_max_ = 27.5º
ω scans	θ_min_ = 1.6º
Absorption correction: multi-scan(CrystalClear; Rigaku, 2008)	*h* = −16→16
*T*_min_ = 0.987, *T*_max_ = 0.991	*k* = −19→19
24346 measured reflections	*l* = −13→13

### Refinement

**Table:**

Refinement on *F*^2^	Secondary atom site location: difference Fourier map
Least-squares matrix: full	Hydrogen site location: inferred from neighbouring sites
*R*[*F*^2^ > 2σ(*F*^2^)] = 0.048	H-atom parameters constrained
*wR*(*F*^2^) = 0.129	*w* = 1/[σ^2^(*F*_o_^2^) + (0.0756*P*)^2^ + 0.2039*P*] where *P* = (*F*_o_^2^ + 2*F*_c_^2^)/3
*S* = 1.07	(Δ/σ)_max_ < 0.001
4655 reflections	Δρ_max_ = 0.22 e Å^−^^3^
275 parameters	Δρ_min_ = −0.28 e Å^−^^3^
Primary atom site location: structure-invariant direct methods	Extinction correction: none

### Special details

**Table:**

Geometry. All e.s.d.'s (except the e.s.d. in the dihedral angle between two l.s. planes) are estimated using the full covariance matrix. The cell e.s.d.'s are taken into account individually in the estimation of e.s.d.'s in distances, angles and torsion angles; correlations between e.s.d.'s in cell parameters are only used when they are defined by crystal symmetry. An approximate (isotropic) treatment of cell e.s.d.'s is used for estimating e.s.d.'s involving l.s. planes.
Refinement. Refinement of *F*^2^ against ALL reflections. The weighted *R*-factor *wR* and goodness of fit *S* are based on *F*^2^, conventional *R*-factors *R* are based on *F*, with *F* set to zero for negative *F*^2^. The threshold expression of *F*^2^ > σ(*F*^2^) is used only for calculating *R*-factors(gt) *etc*. and is not relevant to the choice of reflections for refinement. *R*-factors based on *F*^2^ are statistically about twice as large as those based on *F*, and *R*-factors based on ALL data will be even larger.

### Fractional atomic coordinates and isotropic or equivalent isotropic displacement parameters (Å^2^)

**Table:**

	*x*	*y*	*z*	*U*_iso_*/*U*_eq_	
O1	0.55819 (8)	0.34748 (6)	0.49413 (9)	0.0273 (2)	
O2	0.26913 (7)	0.14553 (6)	0.77917 (8)	0.0227 (2)	
O3	0.20369 (7)	0.09108 (6)	0.55062 (9)	0.0220 (2)	
O4	0.33628 (7)	0.06865 (6)	0.36480 (8)	0.0234 (2)	
N1	0.72197 (8)	0.30131 (7)	0.76057 (10)	0.0197 (2)	
N2	0.64355 (8)	0.29005 (7)	0.66497 (10)	0.0197 (2)	
C1	0.85205 (10)	0.13495 (9)	0.95665 (12)	0.0216 (3)	
H1	0.8091	0.0874	0.9338	0.026*	
C2	0.93150 (10)	0.12422 (9)	1.05376 (12)	0.0231 (3)	
C3	0.94735 (11)	0.04348 (10)	1.11946 (13)	0.0283 (3)	
H3	0.9034	−0.0043	1.1005	0.034*	
C4	1.02660 (12)	0.03530 (11)	1.21027 (14)	0.0342 (4)	
H4	1.0357	−0.0178	1.2532	0.041*	
C5	1.09444 (12)	0.10648 (12)	1.23931 (14)	0.0377 (4)	
H5	1.1488	0.0999	1.3003	0.045*	
C6	1.08098 (11)	0.18525 (12)	1.17846 (14)	0.0351 (4)	
H6	1.1262	0.2320	1.1987	0.042*	
C7	0.99889 (10)	0.19670 (10)	1.08487 (13)	0.0261 (3)	
C8	0.98307 (11)	0.27731 (10)	1.01867 (14)	0.0301 (3)	
H8	1.0270	0.3250	1.0380	0.036*	
C9	0.90514 (10)	0.28610 (9)	0.92784 (13)	0.0253 (3)	
H9	0.8960	0.3397	0.8862	0.030*	
C10	0.83730 (10)	0.21415 (9)	0.89572 (12)	0.0197 (3)	
C11	0.75408 (9)	0.22457 (8)	0.79621 (12)	0.0184 (3)	
C12	0.69637 (10)	0.14964 (9)	0.73107 (12)	0.0214 (3)	
H12A	0.6501	0.1195	0.7883	0.026*	
H12B	0.7457	0.1075	0.6971	0.026*	
C13	0.63207 (10)	0.19680 (8)	0.62438 (12)	0.0192 (3)	
H13	0.6678	0.1887	0.5445	0.023*	
C14	0.51784 (10)	0.16670 (8)	0.60773 (12)	0.0184 (3)	
C15	0.48490 (10)	0.12988 (8)	0.49248 (12)	0.0195 (3)	
H15	0.5323	0.1240	0.4276	0.023*	
C16	0.37999 (10)	0.10182 (8)	0.47523 (11)	0.0184 (3)	
C17	0.30944 (10)	0.11043 (8)	0.57241 (12)	0.0184 (3)	
C18	0.34422 (10)	0.14469 (8)	0.68948 (12)	0.0180 (3)	
C19	0.44879 (10)	0.17348 (8)	0.70697 (12)	0.0191 (3)	
H19	0.4721	0.1970	0.7842	0.023*	
C20	0.29410 (12)	0.19068 (11)	0.89501 (13)	0.0322 (3)	
H20A	0.3559	0.1646	0.9353	0.048*	
H20B	0.2353	0.1862	0.9499	0.048*	
H20C	0.3079	0.2516	0.8776	0.048*	
C21	0.17898 (11)	−0.00027 (9)	0.56046 (13)	0.0262 (3)	
H21A	0.2251	−0.0337	0.5084	0.039*	
H21B	0.1066	−0.0099	0.5328	0.039*	
H21C	0.1886	−0.0188	0.6469	0.039*	
C22	0.40060 (12)	0.06731 (10)	0.25729 (13)	0.0293 (3)	
H22A	0.4284	0.1252	0.2432	0.044*	
H22B	0.3587	0.0490	0.1844	0.044*	
H22C	0.4582	0.0267	0.2716	0.044*	
C23	0.61160 (10)	0.35986 (9)	0.59197 (12)	0.0210 (3)	
C24	0.64295 (11)	0.45014 (9)	0.63730 (13)	0.0256 (3)	
H24A	0.7159	0.4608	0.6191	0.038*	
H24B	0.6341	0.4540	0.7269	0.038*	
H24C	0.5989	0.4934	0.5950	0.038*	

### Atomic displacement parameters (Å^2^)

**Table:**

	*U*^11^	*U*^22^	*U*^33^	*U*^12^	*U*^13^	*U*^23^
O1	0.0283 (5)	0.0300 (5)	0.0229 (5)	−0.0031 (4)	−0.0072 (4)	0.0026 (4)
O2	0.0205 (5)	0.0269 (5)	0.0209 (5)	−0.0017 (4)	0.0014 (4)	−0.0047 (4)
O3	0.0155 (4)	0.0227 (5)	0.0273 (5)	−0.0014 (3)	−0.0041 (4)	−0.0010 (4)
O4	0.0213 (5)	0.0314 (5)	0.0173 (4)	−0.0057 (4)	−0.0026 (4)	−0.0045 (4)
N1	0.0154 (5)	0.0242 (6)	0.0190 (5)	−0.0027 (4)	−0.0040 (4)	−0.0014 (4)
N2	0.0179 (5)	0.0210 (6)	0.0199 (5)	−0.0032 (4)	−0.0055 (4)	−0.0011 (4)
C1	0.0166 (6)	0.0257 (7)	0.0224 (6)	−0.0005 (5)	−0.0005 (5)	−0.0035 (5)
C2	0.0167 (6)	0.0330 (7)	0.0197 (6)	0.0049 (5)	0.0009 (5)	−0.0015 (5)
C3	0.0237 (7)	0.0368 (8)	0.0246 (7)	0.0074 (6)	0.0017 (5)	0.0026 (6)
C4	0.0285 (8)	0.0503 (10)	0.0239 (7)	0.0158 (7)	0.0025 (6)	0.0060 (7)
C5	0.0227 (7)	0.0669 (12)	0.0232 (7)	0.0119 (7)	−0.0052 (6)	0.0027 (7)
C6	0.0193 (7)	0.0589 (10)	0.0266 (7)	−0.0004 (7)	−0.0053 (6)	−0.0035 (7)
C7	0.0164 (6)	0.0400 (8)	0.0218 (6)	0.0012 (6)	−0.0015 (5)	−0.0030 (6)
C8	0.0211 (7)	0.0354 (8)	0.0333 (8)	−0.0074 (6)	−0.0049 (6)	−0.0050 (6)
C9	0.0214 (7)	0.0241 (7)	0.0299 (7)	−0.0021 (5)	−0.0034 (5)	−0.0005 (6)
C10	0.0147 (6)	0.0248 (7)	0.0195 (6)	0.0002 (5)	−0.0010 (5)	−0.0032 (5)
C11	0.0151 (6)	0.0220 (6)	0.0181 (6)	−0.0009 (5)	0.0001 (5)	−0.0016 (5)
C12	0.0186 (6)	0.0219 (7)	0.0234 (6)	0.0004 (5)	−0.0043 (5)	−0.0032 (5)
C13	0.0179 (6)	0.0205 (6)	0.0191 (6)	−0.0011 (5)	−0.0017 (5)	−0.0033 (5)
C14	0.0175 (6)	0.0161 (6)	0.0213 (6)	−0.0009 (5)	−0.0035 (5)	0.0011 (5)
C15	0.0188 (6)	0.0208 (6)	0.0188 (6)	−0.0010 (5)	−0.0004 (5)	−0.0008 (5)
C16	0.0209 (6)	0.0170 (6)	0.0169 (6)	−0.0011 (5)	−0.0045 (5)	0.0000 (5)
C17	0.0154 (6)	0.0172 (6)	0.0222 (6)	−0.0002 (5)	−0.0035 (5)	0.0010 (5)
C18	0.0189 (6)	0.0161 (6)	0.0188 (6)	0.0019 (5)	0.0001 (5)	0.0014 (5)
C19	0.0203 (6)	0.0184 (6)	0.0182 (6)	−0.0005 (5)	−0.0039 (5)	−0.0013 (5)
C20	0.0301 (8)	0.0429 (9)	0.0238 (7)	−0.0045 (6)	0.0039 (6)	−0.0126 (6)
C21	0.0220 (6)	0.0272 (7)	0.0292 (7)	−0.0074 (5)	−0.0013 (5)	0.0017 (6)
C22	0.0327 (8)	0.0358 (8)	0.0194 (6)	−0.0121 (6)	0.0029 (6)	−0.0063 (6)
C23	0.0174 (6)	0.0248 (7)	0.0206 (6)	−0.0017 (5)	−0.0005 (5)	0.0017 (5)
C24	0.0255 (7)	0.0226 (7)	0.0286 (7)	−0.0018 (5)	−0.0016 (6)	0.0014 (5)

### Geometric parameters (Å, °)

**Table:**

O1—C23	1.2292 (16)	C10—C11	1.4685 (17)
O2—C18	1.3642 (16)	C11—C12	1.5040 (18)
O2—C20	1.4294 (16)	C12—C13	1.5424 (18)
O3—C17	1.3769 (15)	C12—H12A	0.9700
O3—C21	1.4255 (16)	C12—H12B	0.9700
O4—C16	1.3699 (15)	C13—C14	1.5155 (17)
O4—C22	1.4204 (16)	C13—H13	0.9800
N1—C11	1.2851 (17)	C14—C15	1.3913 (17)
N1—N2	1.3997 (14)	C14—C19	1.3913 (18)
N2—C23	1.3636 (17)	C15—C16	1.3957 (17)
N2—C13	1.4845 (16)	C15—H15	0.9300
C1—C10	1.3734 (19)	C16—C17	1.3894 (18)
C1—C2	1.4188 (18)	C17—C18	1.3994 (18)
C1—H1	0.9300	C18—C19	1.3953 (18)
C2—C3	1.419 (2)	C19—H19	0.9300
C2—C7	1.421 (2)	C20—H20A	0.9600
C3—C4	1.367 (2)	C20—H20B	0.9600
C3—H3	0.9300	C20—H20C	0.9600
C4—C5	1.405 (2)	C21—H21A	0.9600
C4—H4	0.9300	C21—H21B	0.9600
C5—C6	1.366 (2)	C21—H21C	0.9600
C5—H5	0.9300	C22—H22A	0.9600
C6—C7	1.4175 (19)	C22—H22B	0.9600
C6—H6	0.9300	C22—H22C	0.9600
C7—C8	1.420 (2)	C23—C24	1.5001 (19)
C8—C9	1.3565 (19)	C24—H24A	0.9600
C8—H8	0.9300	C24—H24B	0.9600
C9—C10	1.4210 (18)	C24—H24C	0.9600
C9—H9	0.9300		
			
C18—O2—C20	117.62 (10)	N2—C13—H13	109.1
C17—O3—C21	114.09 (10)	C14—C13—H13	109.1
C16—O4—C22	117.66 (10)	C12—C13—H13	109.1
C11—N1—N2	107.97 (10)	C15—C14—C19	121.04 (11)
C23—N2—N1	120.09 (10)	C15—C14—C13	118.42 (11)
C23—N2—C13	123.58 (10)	C19—C14—C13	120.51 (11)
N1—N2—C13	112.62 (9)	C14—C15—C16	119.29 (12)
C10—C1—C2	121.38 (12)	C14—C15—H15	120.4
C10—C1—H1	119.3	C16—C15—H15	120.4
C2—C1—H1	119.3	O4—C16—C17	114.63 (11)
C1—C2—C3	122.49 (13)	O4—C16—C15	125.07 (12)
C1—C2—C7	118.68 (12)	C17—C16—C15	120.23 (11)
C3—C2—C7	118.82 (12)	O3—C17—C16	119.86 (11)
C4—C3—C2	120.61 (14)	O3—C17—C18	119.92 (11)
C4—C3—H3	119.7	C16—C17—C18	120.07 (11)
C2—C3—H3	119.7	O2—C18—C19	125.51 (11)
C3—C4—C5	120.53 (15)	O2—C18—C17	114.56 (11)
C3—C4—H4	119.7	C19—C18—C17	119.92 (12)
C5—C4—H4	119.7	C14—C19—C18	119.38 (11)
C6—C5—C4	120.36 (14)	C14—C19—H19	120.3
C6—C5—H5	119.8	C18—C19—H19	120.3
C4—C5—H5	119.8	O2—C20—H20A	109.5
C5—C6—C7	120.80 (15)	O2—C20—H20B	109.5
C5—C6—H6	119.6	H20A—C20—H20B	109.5
C7—C6—H6	119.6	O2—C20—H20C	109.5
C6—C7—C8	122.43 (13)	H20A—C20—H20C	109.5
C6—C7—C2	118.86 (14)	H20B—C20—H20C	109.5
C8—C7—C2	118.69 (12)	O3—C21—H21A	109.5
C9—C8—C7	121.31 (13)	O3—C21—H21B	109.5
C9—C8—H8	119.3	H21A—C21—H21B	109.5
C7—C8—H8	119.3	O3—C21—H21C	109.5
C8—C9—C10	120.58 (13)	H21A—C21—H21C	109.5
C8—C9—H9	119.7	H21B—C21—H21C	109.5
C10—C9—H9	119.7	O4—C22—H22A	109.5
C1—C10—C9	119.34 (12)	O4—C22—H22B	109.5
C1—C10—C11	120.81 (11)	H22A—C22—H22B	109.5
C9—C10—C11	119.84 (12)	O4—C22—H22C	109.5
N1—C11—C10	121.18 (11)	H22A—C22—H22C	109.5
N1—C11—C12	114.11 (11)	H22B—C22—H22C	109.5
C10—C11—C12	124.67 (11)	O1—C23—N2	120.02 (12)
C11—C12—C13	102.58 (10)	O1—C23—C24	122.63 (12)
C11—C12—H12A	111.3	N2—C23—C24	117.35 (11)
C13—C12—H12A	111.3	C23—C24—H24A	109.5
C11—C12—H12B	111.3	C23—C24—H24B	109.5
C13—C12—H12B	111.3	H24A—C24—H24B	109.5
H12A—C12—H12B	109.2	C23—C24—H24C	109.5
N2—C13—C14	113.80 (10)	H24A—C24—H24C	109.5
N2—C13—C12	100.80 (9)	H24B—C24—H24C	109.5
C14—C13—C12	114.71 (11)		
			
C11—N1—N2—C23	−166.22 (12)	N1—N2—C13—C12	12.91 (13)
C11—N1—N2—C13	−7.16 (14)	C11—C12—C13—N2	−12.83 (12)
C10—C1—C2—C3	179.06 (12)	C11—C12—C13—C14	−135.52 (11)
C10—C1—C2—C7	−2.04 (19)	N2—C13—C14—C15	125.51 (12)
C1—C2—C3—C4	178.34 (12)	C12—C13—C14—C15	−119.12 (13)
C7—C2—C3—C4	−0.6 (2)	N2—C13—C14—C19	−56.38 (15)
C2—C3—C4—C5	−0.6 (2)	C12—C13—C14—C19	58.98 (15)
C3—C4—C5—C6	1.0 (2)	C19—C14—C15—C16	1.96 (19)
C4—C5—C6—C7	−0.2 (2)	C13—C14—C15—C16	−179.94 (11)
C5—C6—C7—C8	−179.35 (14)	C22—O4—C16—C17	173.08 (11)
C5—C6—C7—C2	−1.0 (2)	C22—O4—C16—C15	−3.84 (18)
C1—C2—C7—C6	−177.61 (12)	C14—C15—C16—O4	176.58 (11)
C3—C2—C7—C6	1.34 (19)	C14—C15—C16—C17	−0.18 (19)
C1—C2—C7—C8	0.84 (19)	C21—O3—C17—C16	83.24 (14)
C3—C2—C7—C8	179.79 (13)	C21—O3—C17—C18	−101.35 (13)
C6—C7—C8—C9	178.77 (13)	O4—C16—C17—O3	−3.74 (16)
C2—C7—C8—C9	0.4 (2)	C15—C16—C17—O3	173.34 (11)
C7—C8—C9—C10	−0.5 (2)	O4—C16—C17—C18	−179.15 (11)
C2—C1—C10—C9	1.98 (19)	C15—C16—C17—C18	−2.06 (18)
C2—C1—C10—C11	−179.40 (11)	C20—O2—C18—C19	9.38 (18)
C8—C9—C10—C1	−0.7 (2)	C20—O2—C18—C17	−171.42 (12)
C8—C9—C10—C11	−179.35 (12)	O3—C17—C18—O2	7.90 (16)
N2—N1—C11—C10	179.66 (10)	C16—C17—C18—O2	−176.70 (10)
N2—N1—C11—C12	−2.55 (14)	O3—C17—C18—C19	−172.85 (11)
C1—C10—C11—N1	162.71 (12)	C16—C17—C18—C19	2.55 (18)
C9—C10—C11—N1	−18.67 (19)	C15—C14—C19—C18	−1.47 (18)
C1—C10—C11—C12	−14.84 (19)	C13—C14—C19—C18	−179.53 (11)
C9—C10—C11—C12	163.78 (12)	O2—C18—C19—C14	178.36 (11)
N1—C11—C12—C13	10.43 (14)	C17—C18—C19—C14	−0.80 (18)
C10—C11—C12—C13	−171.87 (11)	N1—N2—C23—O1	166.45 (11)
C23—N2—C13—C14	−65.56 (16)	C13—N2—C23—O1	9.77 (19)
N1—N2—C13—C14	136.22 (11)	N1—N2—C23—C24	−14.40 (17)
C23—N2—C13—C12	171.12 (11)	C13—N2—C23—C24	−171.08 (11)

### Hydrogen-bond geometry (Å, °)

**Table:**

*D*—H···*A*	*D*—H	H···*A*	*D*···*A*	*D*—H···*A*
C5—H5···O4^i^	0.93	2.48	3.3327 (19)	152
C8—H8···O3^ii^	0.93	2.57	3.4320 (19)	155
C12—H12A···O1^iii^	0.97	2.55	3.3359 (18)	138
C19—H19···O1^iii^	0.93	2.53	3.3073 (18)	141

Symmetry codes: (i) *x*+1, *y*, *z*+1; (ii) *x*+1, −*y*+1/2, *z*+1/2; (iii) *x*, −*y*+1/2, *z*+1/2.
